# Identification and Functional Analysis of Two New Mutant *BnFAD2* Alleles That Confer Elevated Oleic Acid Content in Rapeseed

**DOI:** 10.3389/fgene.2018.00399

**Published:** 2018-09-20

**Authors:** Weihua Long, Maolong Hu, Jianqin Gao, Song Chen, Jiefu Zhang, Li Cheng, Huiming Pu

**Affiliations:** Key Lab of Cotton and Rapeseed (Nanjing) of Ministry of Agriculture, Institute of the Industrial Crops, Jiangsu Academy of Agriculture Sciences, Nanjing, China

**Keywords:** rapeseed (*Brassica napus* L.), *BnFAD2*, single-nucleotide polymorphism, oleic acid, homolog genes, genotyping

## Abstract

Rapeseed (*Brassica napus* L.) is a vital oil crop worldwide. High oleic acid content is a desirable quality trait for rapeseed oil, which makes it more beneficial to human health. However, many germplasm resources with high oleic acid content in rapeseed have not been evaluated with regard to their genotypes, making it difficult to select the best strains with this trait for the breeding of high oleic acid rapeseed variety. This work was to explore the gene-regulation mechanism of this trait using a new super-high oleic acid content (∼85%) line N1379T as genetic material. In this study, the sequences of four homologous *fatty acid desaturase* (*BnFAD2*) genes were compared between super-high (∼85%, N1379T) and normal (∼63%) oleic acid content lines. Results showed that there were two single-nucleotide polymorphisms (SNPs) in *BnFAD2-1* and *BnFAD2-2*, respectively, which led to the amino acid changes (E106K and G303E) in the corresponding proteins. Functional analysis of both genes in yeast confirmed that these SNPs were loss-of-function mutations, thus limiting the conversion of oleic acid to linoleic acid and resulting in the considerable accumulation of oleic acid. Moreover, two specific cleaved amplified polymorphic sequences (CAPS) markers for the two SNPs were developed to identify genotypes of each line in the F_2_ and BC_1_ populations. Furthermore, these two mutant loci of *BnFAD2-1* and *BnFAD2-2* genes were positively associated with elevated oleic acid levels and had a similar effect with regard to the increase of oleic acid content. Taken together, these two novel SNPs in two different *BnFAD2* genes jointly regulated the high oleic acid trait in this special germplasm. The study provided insight into the genetic regulation involved in oleic acid accumulation and highlighted the use of new alleles of *BnFAD2-1* and *BnFAD2-2* in breeding high oleic acid rapeseed varieties.

## Introduction

Rapeseed (*Brassica napus* L.) is one of the most important oil crops across the world. The annual production of rapeseed oil has reached 60–70 million tons, ranks just behind that of soybeans globally (Khan et al., 2018). Similar to other vegetable oils, the fatty acid composition of rapeseed oil is the key trait involved in its utilization mode and range (Napier et al., 2014). After the double-low(low erucic acid and glucosinolates) improvement, rapeseed oil now normally contains ∼7% saturated acids (including palmitic acid C16:0) and stearic acid C18:0), ∼61% oleic acid (C18:1), ∼11% linoleic acid (C18:2), and ∼21% linolenic acid (C18:3) (Sharafi et al., 2015). However, rapeseed oil with higher oleic acid (>75%) has several advantages compared with non-high oleic acid varieties: (1) its anti-oxidative ability provides a longer shelf life, eliminating the need for a hydrogenation process (Browse et al., 1998; Lauridsen et al., 1999; Przybylski et al., 2013); (2) it can decrease low-density lipoprotein levels in humans and then decrease the risk of cardiovascular disease (Chang and Huang, 1998; WHO, 2003); and (3) it is a more cost-effective alternative to olive oil in daily cooking processes, while having a similar function, fatty acid composition, and taste. Therefore, increasing oleic acid content in rapeseed oil has been one of the major objectives for researchers focus on improving rapeseed oil quality.

The fatty acid biosynthesis pathway is a primary metabolic pathway in oil-bearing plants (Ohlrogge and Browse, 1995). Acetyl-CoA is the basic building block of the fatty acid chain and enters the cycle of reduction, dehydration, and reduction to synthesize 16- or 18-carbon products, which are the major (up to 90%) fatty acids of plants. Various desaturases locate in the plastids and the endoplasmic reticulum are responsible for catalyzing these fatty acids to become monounsaturated (palmitoleic acid, C16:1, and C18:1) or polyunsaturated ones (C18:2 and C18:3). Delta-12 fatty acid desaturase 2 (Δ12-FAD2) converts oleic acid precursors to linoleic acid precursors in the lipid biosynthetic pathway (Somerville and Browse, 1991; Ohlrogge and Browse, 1995). The first plant *FAD2* gene was identified in *Arabidopsis* (Okuley et al., 1994) and was soon followed by its discovery in other crops, including soybean (Heppard et al., 1996), sunflower (Hongtrakul et al., 1998), peanut (López et al., 2000), cotton (Pirtle et al., 2001), and maize (Mikkilineni and Rocheford, 2003). The *FAD2* gene family is quite large and diverse in plants (Dehghan and Yarizade, 2014). In rapeseed, there are four homologous *FAD2* genes (*BnFAD2-1*, *BnFAD2-2*, *BnFAD2-3*, and *BnFAD2-4*) that are separately distributed on chromosomes A5, C5, A1, and C1 (also named as *Bna.A5.FAD2*, *Bna.C5.FAD2*, *Bna.A1.FAD2*, and *Bna.C1.FAD2*, respectively). Notably, *BnFAD2-1* and *BnFAD2-2* are constitutively expressed in all tissues, while *BnFAD2-3* and *BnFAD2-4* are specifically expressed in the seeds and roots. There are no introns in the *BnFAD2*-expressing code sequences and they share high identity (>99%) in both their DNA and protein sequences, except for *BnFAD2-3*. This exception is not altogether surprising as *BnFAD2-3* is a pseudogene whose coding sequence is terminated by a stop codon resulting in a frame-shift starting at the 164-bp position (Lee et al., 2013).

The four to six distinct *BnFAD2* gene loci were first found in 1997 (Scheffler et al., 1997). Since the close markers linked to these loci in a segregation population were identified, the possible role of *BnFAD2* manipulation in oleic acid regulation was considered in rapeseed (Schierholt et al., 2000). Although various high oleic acid (>75%) rapeseed germplasms have been created *via* mutagenesis (Auld et al., 1992; Schierholt et al., 2001; Hu et al., 2006; Spasibionek, 2006; Yang et al., 2012), only a few have been evaluated at the molecular level. A quantitative trait locus (QTL) was mapped on chromosome N1 (referring to *BnFAD2-4* on chromosome C1) in a double haploid (DH) population derived from the DSM100 strain (77% C18:1) (Hu et al., 2006). A single-nucleotide mutation from C to T was identified after sequence analysis of *BnFAD2-4*, and the allelic markers developed from this mutation were highly correlated to oleic acid content. A similar study was conducted with the high oleic acid variety SW Hickory (78% C18:1) and a 4-bp insertion in the *BnaA.FAD2.a* sequence (referring to *BnFAD2-1* on chromosome A5) was identified that resulted in misreading and premature termination of translation at the 667–669 bp (TGA) position (Yang et al., 2012). Moreover, an ethyl methanesulfonate (EMS) mutagenized population with varying oleic acid content was also established using the high oleic acid (∼75%) cultivar Cabriolet. Some lines in this population were shown to have the nucleotide deletion in *BnaA.FAD2.a* (again referring to *BnFAD2-1* on chromosome A5) as well as other mutations in *BnaC.FAD2.a* (referring to *BnFAD2-2* on chromosome A1) (Wells et al., 2014). Some other loci (except *BnFAD2*) on genome were also found to have effect on the oleic acid content in rapeseed by the QTL analysis and genome-wide association study (GWAS) methods (Wang et al., 2015; Gacek et al., 2016; Huang et al., 2016;Javed et al., 2016; Qu et al., 2017), but with no further results. In addition, several high oleic acid rapeseed lines have also been created by gene transferring (Peng et al., 2010; Jung et al., 2011; Lee et al., 2016).

We had developed a mutant rapeseed population and found lines with elevated oleic acid content. A stable super-high oleic acid (∼85%) germplasm named N1379T had been screened out. In present study, the *BnFAD2* genes of rapeseed lines with high- and normal-oleic acid content were cloned and two single-nucleotide polymorphisms (SNPs) in *BnFAD2* genes in the high oleic acid rapeseed line N1379T were identified. The functions of these mutational alleles were also confirmed in yeast. Additionally, the association between genotypes and oleic acid contents was explored. This study would enrich the knowledge of genetic mechanisms of high oleic acid content and facilitate breeding high oleic acid varieties in rapeseed.

## Materials and Methods

### Plant Materials

The three double-low rapeseed (*B. napus* L.) lines used in this study were provided by the Jiangsu Academy of Agriculture Sciences (JAAS). N1379T, a stable super-high oleic acid content (∼85%) line, was developed through pedigree selection from an EMS-treated mutagenesis population. The selection procedure was briefly described as follows: plants of the first three generations (M0–M2) from this population underwent open pollination in a greenhouse and lines with the top 50 oleic acid contents were chosen as materials of the next generation. Lines with >80% oleic acid content in generation M3 were performed the microspore culture at the flowering stage and a DH line named N1379T with 85% oleic acid content was selected based on the agronomic traits. Two self-pollinated lines with different genetic backgrounds, 16wh53 and 16wh62, were used as the normal oleic acid content (∼63%) lines.

### Population Development and Fatty Acid Analysis

Two crosses were made to produce the F_1_ generation (N1379T × 16wh53 and 16wh62 × N1379T) at the experimental field of JAAS (Nanjing, China; 32°02′03″N, 118°52′19″E) in the spring of 2014. The three parents and two F_1_s were planted independently at the same site in Nanjing as well as in Wuhan at the research field of the Oil Crops Research Institute of the Chinese Academy of Agricultural Sciences (Wuhan, China; 32°34′57″N, 114°20′11″E) in the autumn of 2014. The two F_2_ populations were constructed by self-pollination of the F_1_ lines from the N1379T × 16wh53 (designated F2-1) and 16wh62 × N1379T (designated F2-2) crosses at the two separate sites in the spring of 2015. At the same time, two backcrossed (BC_1_) populations were produced by crossing two F_1_s (as the female parent) separately with 16wh53 and 16wh62 (designated BC1-1 and BC1-2, respectively) at the two locations.

More than 200 seeds were collected from each population and were used for half-seed analysis following the method outlined by Downey and Harvey (1963). Briefly, for a single seed, one cotyledon inside the seed was cut to detect fatty acid composition, while the other cotyledon containing the embryo was left to germinate in a control room. They were then transferred to the field to continue growing as a single F_2_ or BC_1_ plant. All the materials planted in the field were managed carefully.

### DNA Isolation, RNA Extraction, and cDNA Synthesis

Genomic DNA was extracted from fresh leaves at the seedling stage of the three parent lines as well as each F_1_, F_2_, and BC_1_ line using the protocol described by Sambrook and Russell (2001). DNA concentration was determined for each sample using a Nanodrop 2000 (Thermo Scientific Company, United States) and then diluted to 50 ng/μl in ddH_2_O.

Young seeds of N1379T and 16wh53 were taken from 30-day-old siliques after pollination and put into liquid N_2_. The frozen seeds were grinded into powders and RNA was isolated using a RNA extraction Kit Promega Z3100 (Promega, United States) according to the manufacturer’s instructions. The quality and purity of the RNA was evaluated with a Bioanalyzer 2100 and RNA 6000 Nano Lab Chip Kit (Agilent, Santa Clara, CA, United States). For each sample, about 50 ng of purified RNA was used for cDNA synthesis following the detailed protocol of PrimeScript^TM^ 1st Strand cDNA Synthesis Kit (Takara, Dalian, China). The cDNA products were stored until use.

### SNP Identification

Four *BnFAD2* homolog gene sequences (JN992606, JN992607, JN992608, and JN992609, corresponding to *BnFAD2-1*, *BnFAD2-2*, *BnFAD2-3*, and *BnFAD2-4*), were downloaded from^1^ GenBank. By comparing these sequences in DNAMAN 6.0 (Lynnon Biosoft, United States), the specific primers P1 and P2 (**Table 1**) were designed by Primer 5.0 software (PREMIER Biosoft, United States) and used to amplify all of the homologous *BnFAD2* genes. For the PCR assay, 1 μl of cDNA was used as the template in 20-μl reaction mixtures containing 1× buffer mix with 0.5 U of *pfu* enzyme as well as 0.25 μM of each primer (Transgene, China). The PCR conditions were as follows: initial cycle of 4 min at 95°C; followed by 35 cycles of denaturation at 94°C for 30 s, primer annealing at 59°C for 30 s, and extension at 72°C for 1 min, and finally, a single extension cycle of 10 min at 72°C. The amplification products were size-separated on a 1.5% (M/V) agarose gel. The objective bands (∼1.1 kb) were excised and purified using a DNA Gel Extraction Kit (Transgene, China). The products were then ligated separately into pGEM-T vectors for cloning. About 200 single positive colonies for each template were chosen for sequencing (Tsingke Company, Nanjing, China). All of the *BnFAD2* sequences of N1379T and 16wh53 were arranged into four groups according to previous results (Lee et al., 2013). Four *BnFAD2* homolog genes were downloaded from the reference genome (ZS11^2^). Sequences within the same groups were compared using DNAman 6.0. Each clone was sequenced twice using the forward and reverse primers. Only clones with the same sequences from both primers were analyzed. Nucleotide mutations were recorded only if they appeared more than five times in one group. The DNA sequences were then translated into their amino acids sequences, and a similar comparison was performed with these amino acid sequences.

**Table 1:** Primers used in this study.

Primer name	Sequence (5′-3′)	Purpose
P1	ATGGGTGCAGGTGGAAGAATGCAAG	Cloning all homologous *BnFAD2* genes
P2	ACTTATTGTTGTACCAGAACACACC	
HO10	CGTCTGGGTCATAGCCAACG	Differentiating WT and mutant *BnFAD2-1* alleles
HO11	CGTCTGGGTCATAGCCAACA	
HO12	GACGCTCACGGTCGTTGTAGATG	
HO13	CACCGTTGACAGAGACTTCGG	Differentiating WT and mutant *BnFAD2-2* alleles
HO14	CACCGTTGACAGAGACTTCGA	
HO15	ACACACCTTTCTTCTCACCTTGC	
P3	GCAAGTGTCTCCTCCCTCCAARAAG	Amplifying *BnFAD2-1* gene fragment
P4	AGGCAACTCCTTGGACAGCA	
P5	GCTACGGTCTCTTCCGTTACGGC	Amplifying *BnFAD2-1* gene fragment
P6	CTTGCCTGTCCGGTTCCACATAGATA	


Allelic primers (HO10, HO11, and HO12) were designed to clone the *BnFAD2-1* SNP-containing gene fragments. The HO10–HO12 pair was used for the wild-type (WT) G316, while the HO11–HO12 pair was used for the mutant G316A. Similarly, allelic primers (HO13, HO14, and HO15) were designed for the *BnFAD2-2* SNP, with the HO13–HO15 pair being used for the WT G908, and the HO14–HO15 pair being used for the mutant G908A. The primer sequences are shown in **Table 1**. The genome DNAs from 22 varieties of rapeseed (**Supplementary Table S1**) in China were used as the PCR template. The PCR assay was conducted in the same manner as that described above, except the annealing temperature, which was 59°C in this analysis. The amplification products were size separated on a 1.5% (M/V) agarose gel and then imaged (Universal Hood II, Bio-rad).

### Gene Transformation and Functional Expression in *Saccharomyces cerevisiae*

The WT and corresponding mutant genes were enzyme-digested from the pGEM-T vectors and ligated into the expression vector pYES2 (Invitrogen, United States), which were then transformed into the *S. cerevisiae* strain INVSc1 (Invitrogen) using the electro-stimulation method (Downey and Harvey, 1963). Positive transformants (at least three for each gene) were selected on medium plates lacking uracil (SC-Ura) and were then inoculated in 50-ml SC-Ura liquid media to grow. At the logarithmic phase, the cells were harvested by centrifugation and washed three times with sterile distilled water. The cells were freeze-dried until fatty acid determination.

### Genotyping

To amplify the *BnFAD2-1* or *BnFAD2-2* gene fragments containing the SNP sites, specific primer pairs were designed in Primer 5.0 by utilizing the differences among the four homologous genes. The primer pairs P3–P4 and P5–P6 (**Table 1**) were used to amplify the pure *BnFAD2-1* or *BnFAD2-2* gene fragments, respectively. The PCR conditions were the same as those described above. The restriction endonuclease sites on the prospective PCR products were identified using the online tool NEBcutter V2.0^3^. All of the PCR products were analyzed for cleaved amplified polymorphic sequences (CAPS). Enzymatic digestions using BssS^α^I and HinfI (NEB company) were performed at 37°C in a water-bath for one hour in 20 μl reaction mixtures containing 5 μl of the PCR product, 2 μl of the provided enzyme buffer (10 × ), 0.5 μl of enzyme (10 U), and 12.5 μl ddH_2_O. After digestion, the products were visualized on 2% agarose gels in 1× TAE buffer. When the band type was the same as the WT, its genotype was marked as uppercase AA or BB (for *BnFAD2-1* and *BnFAD2-2*, respectively). When a mutant type was detected, it was marked as lowercase aa or bb. Furthermore, when both genotypes were detected, they were marked as Aa or Bb.

### Fatty Acid Profile Determination in Yeast and Seeds

Determination of the fatty acid profile in the various yeast strains was performed as follows: the yeast powder (0.05 g) of each strain was mixed with 3 ml of KOH–methanol in the 10-ml tubes and incubated at 70°C for 3–5 h. Then, 2 ml of boron trifluoride–methanol solution was added to the mixture, followed by incubation at 70°C for 1.5 h (after adjusting the pH values to 2.0) to achieve methylation. The fatty acid methyl esters (FAMEs) were recovered by mixing with 3-ml *n*-hexane. The upper liquid layer was isolated for gas chromatography (GC) analysis (Agilent 6400, Redwood City, CA, United States). For determination of the fatty acid profile in the half-seed, the cotyledon sample was crushed using a glass rod and added to a 500-μl mixture of ether: petroleum ether (3:2) at room temperature for 4 h. Then, 250 μl of KOH-methanol was added and incubated at room temperature for 2 h. Tubes were shaken for 1 min after 500 μl of H_2_O was added. Subsequently, 100 μl of the supernatant was pipetted out for GC analysis. Fatty acids were identified by comparing their retention times against the FAME standards (Sigma Chemicals Co., St. Louis, MO, United States) separated on the same GC machine. The seeds collected from parental lines and each line of the populations were used for oil content analysis by near-infrared spectroscopy.

### Data Analysis

The data were tabulated in Microsoft Excel and are presented as the means and standard deviations for each diverse rapeseed line (parental, F_1_, F_2_, and BC_1_) at both locations. By following the method of Eshed and Zamir (1996), the additive effect (A) and dominance effect (D) of *BnFAD2-1* and *BnFAD2-2* in F2 populations were calculated as: A = (phenotype value of mutant homozygotes - phenotype value of WT homozygotes)/2; D = (phenotype value of heterozygotes - (phenotype value of mutant homozygotes + phenotype value of WT homozygotes)/2; and the epistasis effect (I) of two genes was: I = [(phenotype value of aabb genotypes + phenotype value of AABB genotypes) - (phenotype value of aa genotypes + phenotype value of bb genotypes)]/2. Comparisons of the oleic acid content of different genotypes were conducted using one-way ANOVA in Graphpad Prism 7 software (Graphpad Prism Software Inc., San Diego, CA, USA). A *p*-value less than 0.05 was considered significant. A box-and-whiskers plot was made to show the distribution (min, first quartile, median, third quartile, and max) of the oleic acid content under different genotypes in Graphpad Prism 7.

## Results

### Identification of Mutations in the *BnFAD2* Genes of the High Oleic Acid Line

**FIGURE 1:**
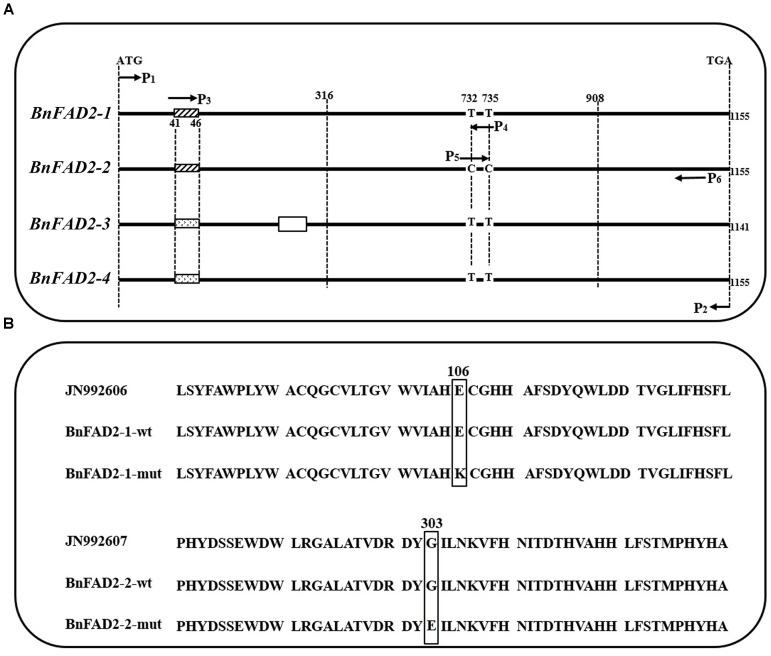
Comparison of the four *BnFAD2* groups and partial alignments of BnFAD2-1 and BnFAD2-2. **(A)** Sequence comparison highlighting the differences between *BnFAD2-1*, *BnFAD2-2*, *BnFAD2-3*, and *BnFAD2-4*. The white box in *BnFAD2-3* indicates the missing 15-bp nucleotide sequence; the differently shaded boxes at 41–46 denote nucleotide difference. The *BnFAD2* homolog genes have characteristic nucleotides at sites 732 and 735. ATG is the start codon; TGA, the stop codon. **(B)** Amino acid sequence alignment of the BnFAD2-1 and BnFAD2-2 peptides. The mutant amino acids are bracketed in square frames. The numbers represent the number of residues from the initiation amino acid Met. JN992606 and JN992607 were used as representative sequences (downloaded from GenBank). wt, wild-type; mut, mutant.

The sequences of cloned *BnFAD2* genes were compared with those from GenBank and from the reference genome. Results showed that the cloned sequences and those from GenBank and reference genome were highly similar and all four *BnFAD2* genes shared identical start and termination coding fragments, which allowed us to design the P1-P2 primer pair to clone all of the homologous *BnFAD2* genes (**Figure 1A**; **Supplementary Table S2**). The positive transformants, 206 and 211, in the T/A clones derived from N1379T and 16wh53, respectively, were sequenced. The *BnFAD2* gene sequences of the lines (N1379T/16wh53) were divided into the four groups corresponding to *BnFAD2-1* (48/55 sequences), *BnFAD2-2* (45/50 sequences), *BnFAD2-3* (40/44 sequences), and *BnFAD2-4* (73/62 sequences). No sequential differences were found for the *BnFAD2-3* and *BnFAD2-4* groups between N1379T and 16wh53. However, two SNPs, G316A in the *BnFAD2-1* group and G908A in the *BnFAD2-2* group, were detected by comparing the two rapeseed lines. These DNA mutations resulted in the amino acid changes E106K and G303E, respectively (**Figure 1B**).

**FIGURE 2:**
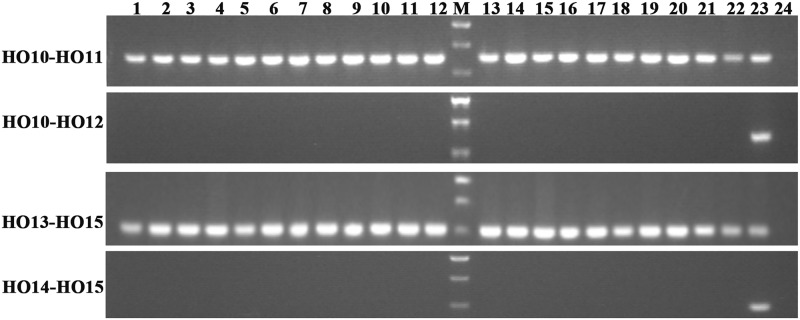
Amplification in registered rapeseed varieties using different primer combinations. Lanes 1–22 refer to the numbered varieties listed in **Supplementary Table S2**. Lane 23, N1379T; Lane 24, H_2_O (negative control). M, DNA marker. There were three standard bands in the M lane representing the 750, 500, and 250 bp band sizes.

Allelic primers were designed to detect these two novel SNPs (G316A and G908A) in 22 registered rapeseed varieties. The HO10–HO12 primer pair (specific to WT G316) amplifies a band for all of the samples, while the HO11–HO12 primer pair (specific to mutant G316A) only amplifies a band from N1379T (**Figure 2**). Similarly, the HO13–HO15 (WT G908) and HO14–HO15 (mutant G908A) primer pairs identified the mutant only in the high oleic acid line N1379T.

### Functional Confirmation of the Mutant *BnFAD2* Genes in *S. cerevisiae*

**FIGURE 3:**
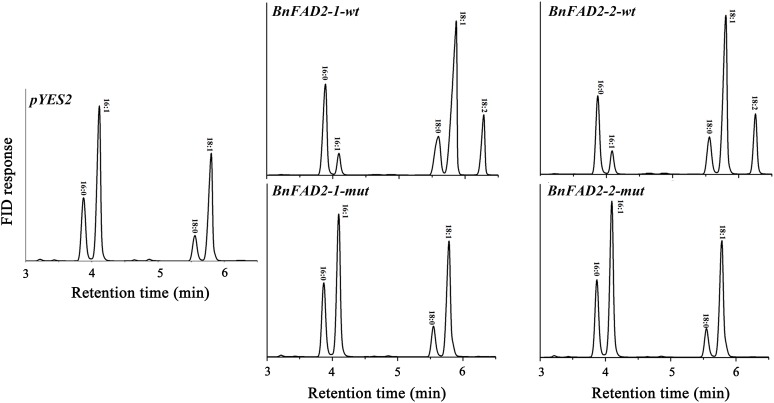
Gas chromatograms showing the fatty acid profiles of *BnFAD2-*transformed yeast. Representative chromatograms highlighting the fatty acid compositions of yeast strains transformed with *pYES2*, *BnFAD2-1-wt*, *BnFAD2-1-mut*, *BnFAD2-2-wt*, and *BnFAD2-2-mut* (top to bottom). The *x* axes represent retention time in minutes, while the *y* axes represent the detector (FID response). 16:0, palmitic acid; 16:1, palmitoleic acid; 18:0, stearic acid; 18:1, oleic acid.

To understand the function of the mutated *BnFAD2-1* and *BnFAD2-2* (designated *BnFAD2-1-mut* and *BnFAD2-2-mut*) genes found in N1379T, these genes and their corresponding WT genes were transformed into the *S. cerevisiae* strain INVSc1. Their function was evaluated by analyzing the fatty acid profile of the yeast strains. The GC analysis indicated that there was a linoleic acid (C18:2) peak detected in the *S. cerevisiae* strains transformed with the *BnFAD2-1-wt* or *BnFAD2-2-wt* gene (9.8 and 10.07% of the total fatty acid content, respectively) (**Figure 3**, details of the fatty acid compositions are shown in **Supplementary Table S3**). However, this peak was not observed in strains transformed with control vector (pYES2), *BnFAD2-1-mut*, or *BnFAD2-2-mut*. Additionally, in the *BnFAD2-1-wt* or *BnFAD2-2-wt* strains, the palmitoleic acid (C16:1) content decreased from 37 to 7% and the oleic acid (C18:1) content increased from 32 to 48%, while the *BnFAD2-1-mut* and *BnFAD2-2-mut* strains had the same fatty acid compositions as the control strains.

### Amplification of Fragments Containing Each SNP and Genotyping

**FIGURE 4:**
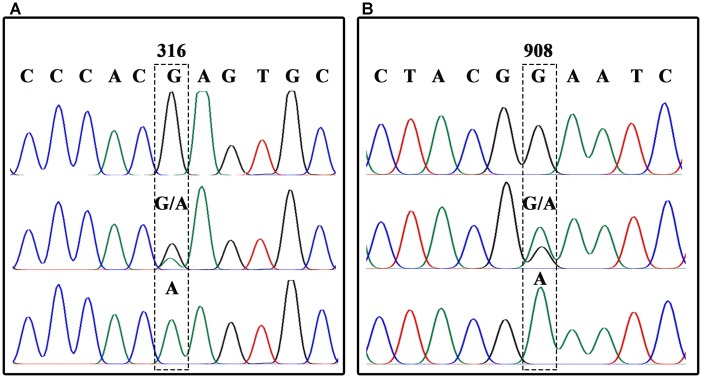
Nucleotide trace map of the mutation site fragments in *BnFAD2-1* **(A)** and *BnFAD2-2* **(B)**. The upper part of the map shows the wild-type sequences, while the middle and lower parts show the hybrid type and mutation type sequences, respectively. The mutant site of each gene is indicated in a dashed frame and the numbers above the frames represent the nucleotide number when counted from the start codon ATG.

In order to analyze each SNP, *BnFAD2* gene fragments containing each site were amplified separately. A sequence comparison detected two fixed variances inside the open reading frames of the four groups, a different 6-bp nucleotide in the 41–46 bp zone for each of the four groups and two distinct nucleotide mutations (C and C) at the 732 and 735 loci in *BnFAD2-2* (**Figure 1A**). Therefore, two primer pairs (P3–P4, P5–P6) specific for *BnFAD2-1* and *BnFAD2-2* were designed according to these differences. The nucleotide trace peaks (**Figure 4**) from the PCR products showed the clear unambiguous line types, indicating that the primer pairs successfully amplified pure fragments of *BnFAD2-1* and *BnFAD2-2*. The sequence differences were easily observed using these sequence peaks. Double peaks were also observed in the hybrid lines.

**FIGURE 5:**
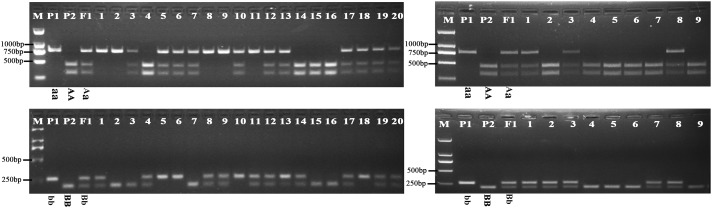
Genotyping of the segregation lines using CAPS markers. The genotype of each line is shown under its enzyme-digested band. The left panel shows the band types for the P1, P2, F1, and F2-1 populations digested by BssS^α^I (upper) and HinfI (lower) restriction enzymes, and no. 1–20 refer to the single lines from the F2-1 population. The right panel shows the band types for the P1, P2, F1, and BC1-1 populations digested by BssS^α^I (upper) and HinfI (lower) restriction enzymes, and no.1-9 refer to the single lines from the BC1-1 population. M, DNA marker; P1, mutant line N1379T; P2, normal line 16wh53. The 60, 80, and 58 bp bands produced by HinfI appear as a single band on the gel because of their very similar lengths; however, these could be differentiated during our analysis. There were three standard bands in the M lane representing the 750, 500, and 250 bp band sizes.

Further analysis showed that the G316A mutation in *BnFAD2-1* mediated the loss of the NssSI recognition site, while the G908A mutation in *BnFAD2-2* mediated loss of the HinfI recognition site. Therefore, these two restriction enzymes could be used to assess genotypes for these mutants. When using the P3-P4 primer pair for BssS^α^I, the WT displayed the 295 bp + 436 bp band-type, while the mutant displayed the 731-bp band-type (**Figure 5**, upper panel). The F_1_ generation, not surprisingly, showed the 295 bp + 436 bp + 731 bp band type. Alternatively, when using the P5–P6 primer pair for HinfI, the WT displayed the 61 bp + 80 bp + 58 bp + 206 bp band type, while the mutant displayed the 61 bp + 80 bp + 264 bp band type (**Figure 5**, lower panel). The F_1_ was again a combination of these, with the 61 bp + 80 bp + 58 bp + 206 bp + 264 bp band type.

### Association Between Mutant *BnFAD2* Alleles and High Oleic Acid Content

The oleic acid content and genotypes were determined for all of the lines in the segregated populations (two F_2_s and two BC_1_s) (**Figure 5**). The parents 16wh53 and 16wh62 producing normal (∼63%) oleic acid content contained the *BnFAD2-1-wt* and *BnFAD2-2-wt* (AABB) genotype. By contrast, the N1379T line had the double *BnFAD2-1-mut* and *BnFAD2-2-mut* (aabb) genotype. Notably, lines with the same genotype as the parental strains had similar oleic acid contents. However, oleic acid content segregation was observed in the F_2_ and BC_1_ populations from the two sites (**Table 2**). There were significant differences for oleic (C18:1), linoleic (C18:2), and linolenic (C18:3) acids between different genotypes in segregation populations, while no differences in oil content existed between genotypes. Generally, F_2_ and BC_1_ lines with more mutant alleles had higher oleic acid content. Indeed, the average oleic acid content of lines with the aabb or AABB genotype in the F_2_ population were ∼85% and ∼63%, respectively, similar to the parental lines. The increase in oleic acid content was companied by a decrease in linoleic and linolenic acids, while the oil contents of genotype lines were without significant change. A similar situation was observed in population F2-2 and BC1-2 (**Supplementary Table S4**).

**Table 2:** Average fatty acid percent and oil content of each genotype in the parental lines and the segregated populations at two locations.

Population	Genotype	Lines	Fatty acid^∗^					Oil
			16:0	18:0	18:1	18:2	18:3	
N1379T (P1)	aabb	8	3.1 ± 0.3	2.1 ± 0.3	85.4 ± 0.5	5.0 ± 0.2	2.51 ± 0.3	41.1 ± 0.5
16WH53 (P2)	AABB	8	3.9 ± 0.4	1.8 ± 0.2	63.5 ± 0.2	20.4 ± 0.2	8.54 ± 0.4	39.9 ± 0.6
F2-1 Nanjing	AABB	16	3.6 ± 0.4 a^∗∗^	1.5 ± 0.2 a	63.1 ± 2.0 a	21.0 ± 1.6 a	7.8 ± 1.2 a	39.3 ± 0.8 a
	AABb	28	4.0 ± 0.4 a	1.9 ± 0.3 a	66.8 ± 1.0 b	18.8 ± 0.9 ab	6.9 ± 0.8 ab	40.6 ± 0.9 a
	AaBB	30	3.0 ± 0.7 a	1.8 ± 0.3 a	66.9 ± 1.6 b	19.3 ± 1.2 ab	6.8 ± 1.3 ab	40.8 ± 1.1 a
	AAbb	15	3.9 ± 0.4 a	1.9 ± 0.2 a	69.8 ± 2.4 bc	17.1 ± 1.4 b	6.3 ± 1.8 ab	41.3 ± 1.2 a
	aaBB	15	3.6 ± 0.4 a	1.7 ± 0.3 a	69.5 ± 1.8 bc	16.2 ± 1.7 b	6.3 ± 1.3 ab	40.9 ± 0.8 a
	AaBb	58	3.7 ± 0.3 a	1.8 ± 0.3 a	73.7 ± 2.0 c	14.4 ± 1.5 bc	5.3 ± 1.1 ab	39.8 ± 1.3 a
	aaBb	27	3.6 ± 0.6 a	1.7 ± 0.3 a	77.6 ± 1.8 d	10.3 ± 1.3 c	4.7 ± 1.2 b	40.2 ± 0.9 a
	Aabb	29	3.8 ± 0.4 a	2.2 ± 0.5 a	78.1 ± 2.3 d	10.5 ± 1.4 c	4.4 ± 1.4 b	41.0 ± 1.4 a
	aabb	14	3.3 ± 0.3 a	2.0 ± 0.3 a	84.9 ± 1.5 e	5.2 ± 1.7 d	2.4 ± 0.3 c	40.6 ± 0.8 a
F2-1 Wuhan	AABB	15	3.9 ± 0.5 a	1.4 ± 0.4 a	63.3 ± 1.9 a	22.3 ± 0.4 a	8.1 ± 1.3 a	38.6 ± 0.8 a
	AABb	26	3.7 ± 0.3 a	1.7 ± 0.2 a	67.1 ± 1.7 b	17.5 ± 1.2 b	7.6 ± 1.2 a	39.2 ± 1.1 a
	AaBB	26	3.4 ± 0.3 ab	1.4 ± 0.1 a	67.0 ± 1.9 b	17.9 ± 0.9 b	7.8 ± 1.2 a	38.7 ± 1.2 a
	AAbb	12	3.3 ± 0.6 ab	1.3 ± 0.2 a	70.0 ± 2.0 b	15.3 ± 1.0 bc	7.1 ± 0.9 a	38.6 ± 0.9 a
	aaBB	13	3.2 ± 0.2 ab	1.6 ± 0.2 a	70.3 ± 1.9 b	16.0 ± 0.9 bc	6.4 ± 0.6 a	40.2 ± 0.8 a
	AaBb	60	3.1 ± 0.5 ab	1.4 ± 0.3 a	74.2 ± 2.0 b	12.6 ± 1.2 c	6.7 ± 1.1 ab	38.8 ± 2.2 a
	aaBb	30	3.0 ± 0.3 b	2.2 ± 0.5 a	78.2 ± 1.9 c	9.9 ± 1.1 d	5.1 ± 0.5 b	39.4 ± 1.7 a
	Aabb	27	2.7 ± 0.6 b	1.9 ± 0.1 a	79.0 ± 2.2 c	8.5 ± 1.0 d	5.0 ± 0.6 b	40.1 ± 1.8 a
	aabb	15	2.9 ± 0.2 b	1.4 ± 0.3 a	85.1 ± 1.4 d	3.6 ± 0.3 e	2.8 ± 0.3 c	39.2 ± 1.6 a
BC1-1 Nanjing	AABB	53	4.0 ± 0.4 a	1.8 ± 0.3 a	63.0 ± 2.0 a	21.0 ± 1.2 a	8.3 ± 1.5 a	39.8 ± 1.4 a
	AABb	52	3.7 ± 0.3 a	2.0 ± 0.2 a	67.5 ± 2.0 b	18.1 ± 1.3 b	7.8 ± 1.1 ab	40.8 ± 1.7 a
	AaBB	54	3.7 ± 0.3 a	1.8 ± 0.2 a	68.4 ± 2.2 b	17.4 ± 1.9 b	6.8 ± 1.4 ab	42.0 ± 1.8 a
	AaBb	55	3.8 ± 0.4 a	1.7 ± 0.2 a	74.0 ± 1.6 c	13.7 ± 1.0 c	5.5 ± 1.2 b	42.3 ± 1.7 a
BC1-1 Wuhan	AABB	56	3.2 ± 0.3 a	2.5 ± 0.2 a	63.2 ± 2.0 a	20.2 ± 1.7 a	8.3 ± 1.4 a	38.7 ± 1.5 a
	AABb	55	3.3 ± 0.2 a	2.3 ± 0.4 a	68.0 ± 1.6 b	18.4 ± 1.0 ab	6.8 ± 1.3 ab	39.1 ± 1.8 a
	AaBB	50	3.8 ± 0.4 a	2.5 ± 0.3 a	69.0 ± 1.9 b	17.8 ± 1.3 ab	7.0 ± 1.5 ab	40.2 ± 2.0 a
	AaBb	57	3.5 ± 0.4 a	2.3 ± 0.4 a	75.0 ± 2.0 c	12.7 ± 1.0 b	5.2 ± 1.1 b	39.6 ± 2.1 a


*^∗^Statistically significant (*p* < 0.05). The comparisons were made within of the corresponding population. ^∗∗^Letters of significance. Two values with the same letter are not statistically different at α = 0.05.*

### Genetic Effects of These Two Mutant Alleles

The genetic effects of additive, dominance, and epistasis in F_2_ populations of these two mutant *BnFAD2* alleles were calculated by using the classic genetic method (**Table 3**). All the effects from each population were positively functioned. The additive effect was the major effect of the alleles, much higher than other two effects (dominance and epistasis effect). The *BnFAD2-1-mut* and *BnFAD2-2-mut* alleles had a very similar genetic effect on the oleic acid content in the four F2 populations. The inheritance of the oleic acid content in N1379T was mainly controlled by the additive effect.

**Table 3:** Genetic effects of the addition, dominance, and epistasis of these two mutant alleles in F_2_ populations.

Genetic effects^∗^	Population			
	F2-1 Nanjing	F2-1 Wuhan	F2-2 Nanjing	F2-2 Wuhan
A-1	5.38	5.53	5.37	5.55
A-2	4.05	3.87	5.48	3.42
D-1	0.95	1.07	1.13	0.92
D-2	0.65	0.72	0.88	1.12
I	1.20	0.92	0.02	0.05


*^∗^A-1 and A-2 denote the additive effects of BnFAD2-1-mut and BnFAD2-2-mut, respectively; D-1 and D-2 signify the dominance effects of BnFAD2-1-mut and BnFAD2-2-mut, respectively; and I indicates the epistasis effect of these two genes.*

### Effects of Each *BnFAD2* Allele on Oleic Acid Content

**FIGURE 6:**
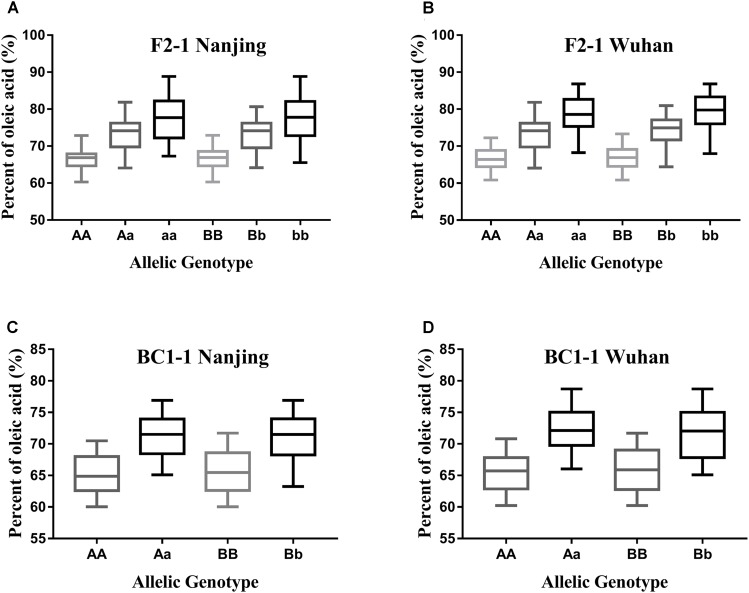
Distribution of the oleic acid content for lines with different allelic genotypes in the segregation populations. Genotype effects mediated by the *BnFAD2-1* (three genotypes: AA, Aa, and aa) and *BnFAD2-2* (three genotypes: BB, Bb, and bb) genes are shown for the F2 population **(A,B)** and BC1 population **(C,D)** at two sites (Nanjing and Wuhan).

As the different alleles segregated into the F_2_ and BC_1_ populations, the effects of each *BnFAD2* allele on the oleic acid content were determined (**Figure 6**). Lines with different alleles of *BnFAD2-1* and *BnFAD2-2* were separated by CAPS markers, and then the oleic acid contents of the same-genotypes lines were calculated in both the F_2_ and BC_1_ populations. Our results showed that both of the *BnFAD2-1* and *BnFAD2-2* had a significant and equal effect on the oleic acid content individually, not just together. For example, compared with the oleic acid content of AA-genotype (homozygous *BnFAD2-1-wt*) lines, the aa-genotype (homozygous *BnFAD2-1-mut*) lines had a 10.68% increase in oleic acid content in the F_2_ population, and the Aa-genotype (heterozygous *BnFAD2-1*) lines had an increase of 6.5%. This is similar to what was observed for the Aa-genotype lines in the BC_1_ population. In the F_2_ populations, the average oleic acid content of the bb-genotype (homozygous *BnFAD2-2-mut*) lines was ∼78%, a 10% increase compared to the BB-genotype (homozygous *BnFAD2-2-wt)*. Furthermore, the same genotype had similar effects in the different populations, with the average oleic acid content of the Aa and Bb genotypes in the F_2_ population being ∼6.69% higher than that of the AA and BB genotypes. The average oleic acid content of the Aa and Bb genotypes in the BC_1_ population was similarly 6.33% higher than that of the AA and BB genotypes.

## Discussion

Beneficial fatty acids facilitate the utilization of rapeseed oil (Napier et al., 2014), and several high oleic acid rapeseed lines have been created by mutagenesis (Auld et al., 1992; Schierholt et al., 2001; Hu et al., 2006; Spasibionek, 2006; Yang et al., 2012). These include N1379T, the stable super-high (∼85%) oleic acid line that was developed by conventional methods. Compared with other vegetable oils, high oleic acid rapeseed oil is a cost-effective alternative for home-cooking. Considering its other features, such as the high oil content (45∼50%) in seeds of rapeseed, it is not surprising that the area of rapeseed cultivation and consumer demand for rapeseed oil have increased (Fu et al., 2016). Unfortunately, our understanding of the genetics and molecular mechanisms underlying high oleic acid traits is limited. In this study, we evaluated mutations in the *BnFAD2* genes in the high oleic acid line (N1379T) to determine the effects of functional loss of *BnFAD2* on oleic acid content.

Notably, we were not the first to identify a relationship between *BnFAD2* mutations and oleic acid content in rapeseed seeds (Hu et al., 2006; Yang et al., 2012); however, aside from *BnFAD2* genes, no other reported QTLs seem to have a connection with oleic acid content. This is particularly true for the transgenic lines with high oleic acid levels (>80%), which were produced by transferring the antisense *BnFAD2* gene fragments into normal oleic acid rapeseed lines (Peng et al., 2010; Jung et al., 2011; Lee et al., 2016). Our results confirmed that the high oleic acid trait in N1379T was controlled by two mutant *BnFAD2* genes (*BnFAD2-1* and *BnFAD2-2*). To our knowledge, this is the first study to provide genetic and molecular evidences of two-gene-controlled high oleic acid trait in rapeseed.

Although it is not altogether surprising that mutations in plant *FAD2* reduce its enzymatic activity and enhance oleic acid content, not all mutations would result in these changes. The position of the mutation in the *BnFAD2* gene is, therefore, very important. Δ12-FAD2 is a trans-membrane protein with three conserved histidine-rich motifs (also called histidine boxes, H-boxes), which form the active center of the enzyme (Shanklin et al., 1994). Different plant species, especially oilseed crops, have multiple *FAD2* genes/proteins with highly similar sequences and structures (Dehghan and Yarizade, 2014). For rapeseed, the three H-boxes in BnFAD2 are 105-HECGHHAF-111, 137-WKYSHRRHH, and 315-HVAHHLFS-323 (Tanhuanpää et al., 1998). Mutations that occur near the H-boxes have a high probability of affecting BnFAD2 enzyme activity. In the present study, one of the detected mutations was E106K in BnFAD2-1, which located within the first H-box. The other mutation site was G303E in BnFAD2-2, near the third H-box motif. Therefore, it is not surprising that these SNP mutations resulted in loss of BnFAD2 function. However, it is not a requirement for the SNP mutation to occur inside or near the H-boxes to be effective. In fact, a series of SNPs were created in a mutagenic oleic acid-enhanced soybean population in which most of the mutations were not in or near the H-boxes (Wells et al., 2014). It was demonstrated that the Ser-185 of GmFAD2-1, while being outside of the H-boxes, played a key role in regulating post-translational modifications that directly affected FAD2-1 enzyme activity (Tang et al., 2005). In the present study, Ser-185 was also not in the H-boxes of the BnFAD2 enzymes. While the investigation of *BnFAD2* regulatory elements inside and/or outside of the genes was outside the scope of this study, future work should explore this regulation in order to more fully explain the relationship between gene mutation and the observed elevation in oleic acid content (Xiao et al., 2014).

It was important to verify the sequences and function of WT and mutant *BnFAD2-1* and *BnFAD2-2*. Therefore, we transformed the genes into yeast, which have been widely used for the functional characterization of FADs. *AtFAD2* was the first *FAD* gene to be functionally expressed in *S. cerevisiae* and resulted in a successful linoleic acid production (Covello and Reed, 1996). This not only confirmed desaturase activity but also proved that yeast could be a suitable host for functional oilseed gene expression. Since then, many *FAD2* genes have been cloned from different oilseeds (e.g., peanut, cotton, sunflower, soybean, olive, etc.) and tested using yeast (Jung et al., 2000; Martínez-Rivas et al., 2001; Pirtle et al., 2001; Hernandez et al., 2005; Tang et al., 2005). In fact, BnFAD2-1 and BnFAD2-2 in WT were also previously confirmed to have catalytic activity in yeast (Xiao et al., 2010; Lee et al., 2013). In our investigation, both the WT and corresponding mutant gene were expressed in yeast to identify mutation-mediated changes in enzyme function. Our results showed that both mutant genes were non-functional and could not catalyze oleic acid to linoleic acid. Furthermore, it showed that both BnFAD2-1 and BnFAD2-2 contributed equally to linoleic acid formation as similar levels were observed in yeast for both genes. Yeast strains transformed with *BnFAD2-1-mut* and *BnFAD2-2-mut* genes had similar fatty acid compositions to pYES2 strains, indicating these two mutant genes were not functional. Meanwhile, a decrease in palmitoleic acid (C16:1) and increases in oleic (C18:1) and linoleic (C18:2) acid were observed in the *BnFAD2-1* and *BnFAD2-2* strains, possibly because these two genes synthesized linoleic acid (C18:2) by catalyzing oleic acid (C18:1), which activated the flow from palmitoleic acid (C16:1) to oleic acid (C18:1). With the palmitoleic acid (C16:1) continued to be processed to form oleic acid (C18:1) in the rapeseed, oleic acid would accumulate, because the mutant *BnFAD2-1* and *BnFAD2-2* could not convert this to linoleic acid (C18:2). Although this work highlighted a definitive role for these mutated sequences in the observed increase in oleic acid, questions still remained concerning these genes. For example, additional work was required to determine the contribution of each *BnFAD2* in the accumulation of oleic acid (C18:1) in rapeseed seeds and whether these genes compensate for each other. Furthermore, future studies should determine if the plant can continue to grow if all of the *BnFAD2* genes lose their function at the same time as well as identify other biological pathways oleic acid (C18:1) participates in (Guan et al., 2012). While our work provided a solid foundation for continued investigation, answering these outstanding questions would assist in further exploring the roles of C18:1 in rapeseed development.

Our results proved that the mutations were in the constitutive-expressing *BnFAD2*, which were in accordance with the significant increase of oleic acid content in both vegetable tissues and seeds of N1379T (Jianqin et al., 2012; Lee et al., 2013). Fatty acids are essential components of plant membranes, and increased oleic acid level will cause a concomitant decrease in polyunsaturated fatty acids. This will limit the general mobilization of membranes inside tissues under low temperatures (Upchurch, 2008). For example, a FAD2 mutant in *Arabidopsis* failed to undergo normal growth during development at low temperatures (Miquel et al., 1993; Miquel and Browse, 1994). Furthermore, another report suggested that high oleic acid expression in rapeseed would likely be accompanied with reduced yield and lower germination rates (Schierholt and Becker, 2001, 2011). Thus, although it was beyond the scope of the present study, a comprehensive exploration was required to evaluate the effects of increased oleic acid on the yield of N1379T.

Ultimately, one of the goals of this work was to detect genetic markers linked to higher oleic acid levels and utilize these markers to improve breeding decisions. To perform marker-assisted selection (MAS), the homologous and heterozygous genotypes must be clearly distinguishable. It is particularly difficult to design specific primers to identify and amplify the mutant SNP sites in *BnFAD2* genes, as they have very similar DNA sequences (Lee et al., 2013). In this study, we developed CAPS markers for both these SNP sites and successfully genotyped lines using PCR and electrophoresis. The CAPS markers were stable and can unambiguously differentiate the parent and hybrid genotypes, allowing for mass selection. Moreover, the two mutated *BnFAD2* genes are located on different chromosomes (A5 and C5), suggesting that they have no linkage effects on each other. Our genotyping system would allow candidate lines with the desired allelic genes to be screened *via* CAPS marker-driven PCR, making it easy to select high oleic acid-expressing rapeseed varieties for breeding. In addition, genetic effect analysis revealed that the additive effect contributed much more to oleic acid content than the other two effects (dominance and epistasis effect). As the additive effect is the inheritable factor, the trait of high oleic acid content in N1379T can be easily transferred to other varieties and utilized in breeding.

## Conclusion

In conclusion, we demonstrated that two separate SNPs in the homologous *BnFAD2-1* and *BnFAD2-2* genes induced loss of enzyme function of both enzymes and were responsible for the super-high oleic acid (85%) content in N1379T. Furthermore, during our investigation we designed CAPS markers to effectively detect the genotypes of these alleles, thus providing a MAS system to use for the selection of this trait. This study provided a foundation on using N1379T as a new valuable trait resource for developing the high oleic acid rapeseed varieties in future.

## Author Contributions

WL designed the experiments, processed the molecular biology and data analysis, and wrote the manuscript. JG determined the fatty acids profiles. MH did the sequences comparison and designed the primers. SC and LC processed part of PCR assays. JZ revised the manuscript. HP selected the germplasm, developed the populations, took part in the experimental design, and finalized the manuscript.

## Conflict of Interest Statement

The authors declare that the research was conducted in the absence of any commercial or financial relationships that could be construed as a potential conflict of interest.

## Footnotes

1 http://www.ncbi.nlm.nih.gov/genbank/

2 http://brassicadb.org/brad/

3 http://nc2.neb.com/NEBcutter2/

## Supplementary Material

TABLE S1 Rapeseed varieties tested.

TABLE S2 Comparison of the cloned *BnFAD2* homolog gene sequences with those downloaded from GenBank and with the reference genome.

TABLE S3 Percentage of each fatty acid in *Saccharomyces cerevisiae* transferred with different *BnFAD2* genes.

TABLE S4 Average fatty acid and oil content percentages of each genotype in the parental lines and the segregated populations (F2-2 and BC1-2) at two locations.
