# Supplementary material for: Identification and Functional Analysis of Two New Mutant BnFAD2 Alleles That Confer Elevated Oleic Acid Content in Rapeseed
Source: Front Genet. 2018 Sep 20;9:399. doi: 10.3389/fgene.2018.00399 (PMC6158388; doi:10.3389/fgene.2018.00399)
Supplement: TABLE S1 — Rapeseed varieties tested. [file Table_1.docx]

Supplementary Material 1

**Identification and Functional Analysis of Two New Mutant *BnFAD2* Alleles that Confer Elevated Oleic Acid Content in Rapeseed**

**Weihua Long^1^, Maolong Hu^1^, Jianqin Gao^1^, Song Chen^1^, Jiefu Zhang^1^, Cheng Li, Huiming Pu^1*^**

**^*^ Correspondence:**

Prof. Huiming Pu

E-mail: [puhuiming@126.com](mailto:puhuiming@126.com)

**Supplementary Table 1.** Rapeseed varieties tested.

| No. | Variety name | Origin |
| --- | --- | --- |
| 1 | Ningza No21 | Jiangsu, China |
| 2 | Ningza 1818 | Jiangsu, China |
| 3 | Zhongshuang No11 | Hubei, China |
| 4 | Suyou No4 | Jiangsu, China |
| 5 | Huyou No17 | Shanghai, China |
| 6 | Yangyou No6 | Jiangsu, China |
| 7 | Wanyou No20 | Anhui, China |
| 8 | Guiza No5 | Guizhou, China |
| 9 | Shanyou 17 | Shanxi, China |
| 10 | Chuanyou 41 | Sichuan, China |
| 11 | Zhongyouza No12 | Hubei, China |
| 12 | Huayouza No9 | Hubei, China |
| 13 | Fengyou 737 | Hunan, China |
| 14 | Qingyou No10 | Shanxi, China |
| 15 | Zheshuang No3 | Zhejiang, China |
| 16 | Ganyouza No7 | Jiangxi, China |
| 17 | Fengyou 520 | Hunan, China |
| 18 | Qingyou No7 | Shanxi, China |
| 19 | Zhongshuang No9 | Hubei, China |
| 20 | Zheyou 50 | Zhejiang, China |
| 21 | Yangguang 2009 | Hubei, China |
| 22 | Yuhuang No4 | Chongqing, China |
| 23 | N1379T | Jiangsu, China |
| 24 | H_2_O (Negative control) | / |
