# Supplementary material for: Identification and Functional Analysis of Two New Mutant BnFAD2 Alleles That Confer Elevated Oleic Acid Content in Rapeseed
Source: Front Genet. 2018 Sep 20;9:399. doi: 10.3389/fgene.2018.00399 (PMC6158388; doi:10.3389/fgene.2018.00399)
Supplement: TABLE S2 — Comparison of the cloned BnFAD2 homolog gene sequences with those downloaded from GenBank and with the reference genome. [file Table_2.docx]

Supplementary Material 2

**Identification and Functional Analysis of Two New Mutant *BnFAD2* Alleles that Confer Elevated Oleic Acid Content in Rapeseed**

**Weihua Long^1^, Maolong Hu^1^, Jianqin Gao^1^, Song Chen^1^, Jiefu Zhang^1^, Cheng Li, Huiming Pu^1*^**

**^*^ Correspondence:**

Prof. Huiming Pu

E-mail: [puhuiming@126.com](mailto:puhuiming@126.com)

**Supplementary Table 2.** Comparison of the cloned *BnFAD2* homologue gene sequences with those downloaded from GenBank and with the reference genome.

Note: ZS11-A05, ZS11-C05, ZS11-A01 and ZS11-C01 are the *BnFAD2* homologue sequences (located on the chrA5, chrC5, chrA1 and chrC1, respectively) from the reference genome of the variety ZS11.
