# Supplementary material for: Identification and Functional Analysis of Two New Mutant BnFAD2 Alleles That Confer Elevated Oleic Acid Content in Rapeseed
Source: Front Genet. 2018 Sep 20;9:399. doi: 10.3389/fgene.2018.00399 (PMC6158388; doi:10.3389/fgene.2018.00399)
Supplement: TABLE S3 — Percentage of each fatty acid in Saccharomyces cerevisiae transferred with different BnFAD2 genes. [file Table_3.docx]

Supplementary Material 3

**Identification and Functional Analysis of Two New Mutant *BnFAD2* Alleles that Confer Elevated Oleic Acid Content in Rapeseed**

**Weihua Long^1^, Maolong Hu^1^, Jianqin Gao^1^, Song Chen^1^, Jiefu Zhang^1^, Cheng Li, Huiming Pu^1*^**

**^*^ Correspondence:**

Prof. Huiming Pu

E-mail: [puhuiming@126.com](mailto:puhuiming@126.com)

**Supplementary Table 3.** Percentage of each fatty acid in *Saccharomyces cerevisiae* transferred with different *BnFAD2* genes.

| Fatty acid* | Strains | | | | |
| --- | --- | --- | --- | --- | --- |
|  | *pYES2* | *BnFAD2-1-wt* | *BnFAD2-1-mut* | *BnFAD2-2-wt* | *BnFAD2-2-mut* |
| 16:0 | 19.78 | 20.68 | 20.03 | 22.73 | 19.87 |
| 16:1 | 37.26 | 7.47 | 40.43 | 7.79 | 38.37 |
| 18:0 | 8.62 | 11.75 | 7.21 | 9.58 | 7.74 |
| 18:1 | 32.45 | 48.24 | 31.11 | 47.69 | 32.20 |
| 18:2 | 0 | 9.80 | 0 | 10.07 | 0 |

* 16:0, palmitic acid; 16:1, palmitoleic acid; 18:0, stearic acid; 18:1, oleic acid; 18:2, linoleic acid.
