# Supplementary material for: Identification and Functional Analysis of Two New Mutant BnFAD2 Alleles That Confer Elevated Oleic Acid Content in Rapeseed
Source: Front Genet. 2018 Sep 20;9:399. doi: 10.3389/fgene.2018.00399 (PMC6158388; doi:10.3389/fgene.2018.00399)
Supplement: TABLE S4 — Average fatty acid and oil content percentages of each genotype in the parental lines and the segregated populations (F2-2 and BC1-2) at two locations. [file Table_4.docx]

Supplementary Material 4

**Identification and Functional Analysis of Two New Mutant *BnFAD2* Alleles that Confer Elevated Oleic Acid Content in Rapeseed**

**Weihua Long^1^, Maolong Hu^1^, Jianqin Gao^1^, Song Chen^1^, Jiefu Zhang^1^, Cheng Li, Huiming Pu^1*^**

**^*^ Correspondence:**

Prof. Huiming Pu

E-mail: [puhuiming@126.com](mailto:puhuiming@126.com)

**Supplementary Table 4.** Average fatty acid and oil content percentages of each genotype in the parental lines and the segregated populations (F2-2 and BC1-2) at two locations.

| Population | Genotype | Lines | Fatty acid |  |  |  |  | Oil |
| --- | --- | --- | --- | --- | --- | --- | --- | --- |
|  |  |  | 16:0 | 18:0 | 18:1 | 18:2 | 18:3 |  |
| N1379T (P1) | aabb | 8 | 3.1 ± 0.3 | 2.1 ± 0.2 | 85.4 ± 0.3 | 5.0 ± 0.2 | 2.5 ± 0.3 | 41.1 ± 0.5 |
| 16WH62 (P3) | AABB | 8 | 3.6 ± 0.4 | 1.6 ± 0.1 | 63.6 ± 0.3 | 21.6 ± 0.6 | 7.4 ± 0.4 | 42.3 ± 0.6 |
|  |  |  |  |  |  |  |  |  |
| F2-2 Nanjing | AABB | 14 | 3.8 ± 0.5 a* | 2.0 ± 0.3 a | 63.8 ± 1.2 a | 22.9 ± 1.3 a | 7.6 ± 1.2 a | 43.2 ± 1.5 a |
|  | AABb | 27 | 3.7 ± 0.4 a | 2.0 ± 0.4 a | 68.6 ± 1.5 b | 17.3 ± 2.0 b | 6.8 ± 1.4 ab | 42.6 ± 0.9 a |
|  | AaBB | 28 | 3.6 ± 0.4 ab | 1.9 ± 0.3 a | 69.0 ± 1.8 b | 17.1 ± 1.5 b | 6.4 ± 1.9 ab | 42.8 ± 1.3 a |
|  | AAbb | 13 | 3.7 ± 0.4 ab | 2.0 ± 0.3 a | 72.1 ± 1.8 bc | 13.9 ± 1.2 c | 6.5 ± 1.1 ab | 40.3 ± 1.8 a |
|  | aaBB | 13 | 3.8 ± 0.2 a | 1.9 ± 0.3 a | 71.6 ± 1.8 bc | 14.6 ± 0.9 cd | 6.3 ± 0.9 ab | 41.9 ± 0.8 a |
|  | AaBb | 56 | 3.7 ± 0.4 ab | 1.9 ± 0.2 a | 75.2 ± 2.2 c | 11.9 ± 1.5 d | 5.4 ± 0.9 ab | 39.5 ± 2.4 a |
|  | aaBb | 25 | 3.1 ± 0.3 ab | 1.9 ± 0.3 a | 79.7 ± 1.8 d | 10.3 ± 1.0 de | 4.1 ± 0.8 b | 41.5 ± 1.2 a |
|  | Aabb | 27 | 3.0 ± 0.3 b | 1.8 ± 0.3 a | 79.8 ± 1.9 d | 9.3 ± 1.5 de | 4.1 ± 0.9 b | 40.6 ± 1.2 a |
|  | aabb | 10 | 3.0 ± 0.2 b | 1.7 ± 0.2 a | 85.4 ± 1.4 e | 5.1 ± 1.2 e | 2.6 ± 0.5 c | 42.1 ± 0.9 a |
|  |  |  |  |  |  |  |  |  |
| F2-1 Wuhan | AABB | 13 | 3.7 ± 0.3 a | 1.7 ± 0.4 a | 63.9 ± 1.7 a | 21.7 ± 1.5 a | 8.1 ± 1.5 a | 39.6 ± 1.5 a |
|  | AABb | 22 | 3.7 ± 0.5 a | 1.9 ± 0.3 a | 68.7 ± 1.8 b | 17.5 ± 1.2 b | 7.8 ± 1.3 a | 41.2 ± 1.5 a |
|  | AaBB | 24 | 3.8 ± 0.4 a | 1.8 ± 0.2 a | 68.7 ± 2.0 b | 18.0 ± 1.0 b | 6.0 ± 1.2 a | 40.7 ± 1.7 a |
|  | AAbb | 10 | 3.8 ± 0.4 a | 2.3 ± 0.3 a | 71.9 ± 1.9 bc | 15.5 ± 1.0 bc | 6.7 ± 1.0 a | 41.9 ± 1.9 a |
|  | aaBB | 11 | 3.7 ± 0.5 a | 2.3 ± 0.2 a | 72.2 ± 1.9 c | 14.8 ± 0.9 bc | 6.2 ± 0.7 a | 40.8 ± 1.4 a |
|  | AaBb | 58 | 3.3 ± 0.3 ab | 2.3 ± 0.4 a | 75.6 ± 2.2 c | 11.9 ± 1.2 c | 5.3 ± 1.2 b | 40.7 ± 2.4 a |
|  | aaBb | 28 | 2.8 ± 0.4 b | 2.2 ± 0.4 a | 80.0 ± 2.2 cd | 9.3 ± 1.2 d | 4.3 ± 1.2 c | 41.1 ± 1.6 a |
|  | Aabb | 25 | 2.6 ± 0.5 b | 1.9 ± 0.2 a | 79.6 ± 2.0 d | 9.7 ± 0.9 d | 3.6 ± 0.9 c | 40.7 ± 2.1 a |
|  | aabb | 12 | 2.8 ± 0.3 b | 2.3 ± 0.5 a | 85.6 ± 1.2 e | 4.7 ± 0.8 e | 2.8 ± 0.4 c | 41.2 ± 1.8 a |
|  |  |  |  |  |  |  |  |  |
| BC1-1 Nanjing | AABB | 51 | 3.8 ± 0.4 a | 2.0 ± 0.3 a | 64.1 ± 1.8 a | 21.5 ± 1.2 a | 8.0 ± 0.7 a | 41.8 ± 1.8 a |
|  | AABb | 53 | 3.7 ± 0.5 a | 2.1 ± 0.2 a | 69.3 ± 2.1 b | 16.5 ± 1.7 a | 6.7 ± 1.4 a | 40.9 ± 1.9 a |
|  | AaBB | 52 | 3.7 ± 0.2 a | 2.5 ± 0.4 a | 70.5 ± 2.1 b | 15.7 ± 1.2 a | 6.6 ± 1.0 a | 43.0 ± 2.1 a |
|  | AaBb | 53 | 3.4 ± 0.4 a | 2.3 ± 0.3 a | 75.3 ± 1.9 c | 12.3 ± 1.6 b | 5.3 ± 0.7 a | 41.3 ± 2.2 a |
|  |  |  |  |  |  |  |  |  |
| BC1-1 Wuhan | AABB | 54 | 3.8 ± 0.4 a | 2.2 ± 0.3 a | 64.0 ± 1.7 a | 20.5 ± 0.7 a | 7.8 ± 1.3 a | 41.4 ± 2.0 a |
|  | AABb | 50 | 3.7 ± 0.3 a | 2.4 ± 0.3 a | 70.0 ± 1.8 b | 17.4 ± 0.9 b | 6.9 ± 1.2 a | 40.9 ± 2.3 a |
|  | AaBB | 48 | 3.4 ± 0.5 a | 2.2 ± 0.4 a | 69.8 ± 1.8 b | 16.8 ± 1.4 b | 6.3 ± 1.1 a | 41.2 ± 2.3 a |
|  | AaBb | 55 | 3.3 ± 0.4 a | 2.5 ± 0.4 a | 75.6 ± 2.1 c | 11.9 ± 1.0 c | 5.3 ± 1.2 a | 40.5 ± 2.2 a |

* Statistically significance (*p* < 0.05). The comparisons were made within of the corresponding populations.
